# Cancer vaccines: an update on recent achievements and prospects for cancer therapy

**DOI:** 10.1007/s10238-024-01541-7

**Published:** 2024-12-25

**Authors:** Arezki Chekaoui, Mariangela Garofalo, Beata Gad, Monika Staniszewska, Jacopo Chiaro, Katarzyna Pancer, Aleksander Gryciuk, Vincenzo Cerullo, Stefano Salmaso, Paolo Caliceti, Aleksander Masny, Magdalena Wieczorek, Sari Pesonen, Lukasz Kuryk

**Affiliations:** 1 Department of Virology, National Institute of Public Health NIH–National Research Institute, Warsaw, Poland; 2 Department of Pharmaceutical and Pharmacological Sciences, University of Padua, Padua, Italy; 3 Centre for Advanced Materials and Technologies, Warsaw University of Technology, Warsaw, Poland; 4 Valo Therapeutics Oy, Helsinki, Finland; 5 Drug Research Program (DRP), ImmunoViroTherapy Lab (IVT), Division of Pharmaceutical Biosciences, Faculty of Pharmacy, University of Helsinki, Helsinki, Finland; 6 Helsinki Institute of Life Science (HiLIFE) University of Helsinki, Helsinki, Finland; 7 Translational Immunology Program (TRIMM), Faculty of Medicine, University of Helsinki, Helsinki, Finland; 8 Digital Precision Cancer Medicine Flagship (iCAN), University of Helsinki, Helsinki, Finland; 9 Department of Molecular Medicine and Medical Biotechnology and CEINGE, University Federico II of Naples, Naples, Italy

**Keywords:** cancer vaccine, cancer immunity, immunotherapy, cancer vaccine platforms, tumour resistance

## Abstract

Decades of basic and translational research have led to a momentum shift in dissecting the relationship between immune cells and cancer. This culminated in the emergence of breakthrough immunotherapies that paved the way for oncologists to manage certain hard-to-treat cancers. The application of high-throughput techniques of genomics, transcriptomics, and proteomics was conclusive in making and expediting the manufacturing process of cancer vaccines. Using the latest research technologies has also enabled scientists to interpret complex and multiomics data of the tumour mutanome, thus identifying new tumour-specific antigens to design new generations of cancer vaccines with high specificity and long-term efficacy. Furthermore, combinatorial regimens of cancer vaccines with immune checkpoint inhibitors have offered new therapeutic approaches and demonstrated impressive efficacy in cancer patients over the last few years. In the present review, we summarize the current state of cancer vaccines, including their potential therapeutic effects and the limitations that hinder their effectiveness. We highlight the current efforts to mitigate these limitations and highlight ongoing clinical trials. Finally, a special focus will be given to the latest milestones expected to transform the landscape of cancer therapy and nurture hope among cancer patients.

## Introduction

Cancer is characterized by the uncontrolled growth of cells that can develop in any part of the body, and if left untreated, may spread to other organs [1]. Despite tremendous diagnostic and therapeutic advancements, cancer is still one of the most dangerous and deadliest diseases in the world [2, 3]. Researchers worldwide are joining their efforts to develop new methods and strategies to address this unmet medical need. For a long time, chemotherapy and radiation have constituted raw materials for oncologists to treat and palliate cancer patients [4, 5]. These therapeutics saved lives and improved patient survival and quality of life [6]. However, the side effects of these therapies during the treatment journey (physical and physiological changes, lack of specificity-differentiation between normal and cancerous cells and effectiveness) have prompted the scientific community to develop new approaches that provide more protection for patients and fill the gaps of these conventional therapies [7, 8].

The immune system is a sophisticated and powerful defence mechanism that safeguards the body against invading pathogens such as viruses and bacteria [9, 10]. It also protects us against the internal mutations of certain cells that may threaten our body systems [11]. Decades of basic research and clinical trials have been critical to elucidate the relationship between the immune system and cancer cells. Exploring these data and bringing them from bench to bedside has led to the emergence of a new therapeutic option called immunotherapy [12]. Currently, immunotherapy offers many alternatives to physicians for managing cancer and subsequently broadens the horizon of curative strategies for better patient care [13]. However, despite all this success, some major limitations still be linked to these immunotherapies (i.e. CAR-T-cell therapy), such as cytokine release syndrome, neurologic toxicities, poor infiltration, weak trafficking, and limited potency [14].

Vaccines are potent tools for treating and preventing infectious and lethal diseases [15, 16]. Throughout history, they have saved and continue to save billions of lives, and the recent outbreak of the COVID-19 pandemic, which turned the world upside down, is the best evidence of their potential as a therapeutic option [17]. The scientific principle behind using vaccines is to take advantage of the human immune system; they are designed to teach and train the immune system to combat and expel pathogens, similar to how these microorganisms are encountered within the body in real scenarios [18]. In fact, cancer vaccines—one of the multiple categories of immunotherapy—are not an exception; with some particularities compared to conventional vaccines, they are designed to educate immune cells that cancerous cells are intruders that need to eliminate [19].

In this review, we will shed light on cancer vaccine treatments and underline their potential role, with emphasis on recent clinical trials and FDA approval. We will discuss the different obstacles that hinder their effectiveness and highlight the current efforts to address these limitations and make vaccines more effective.

## Principles of cancer immunity

**Fig. 1:**
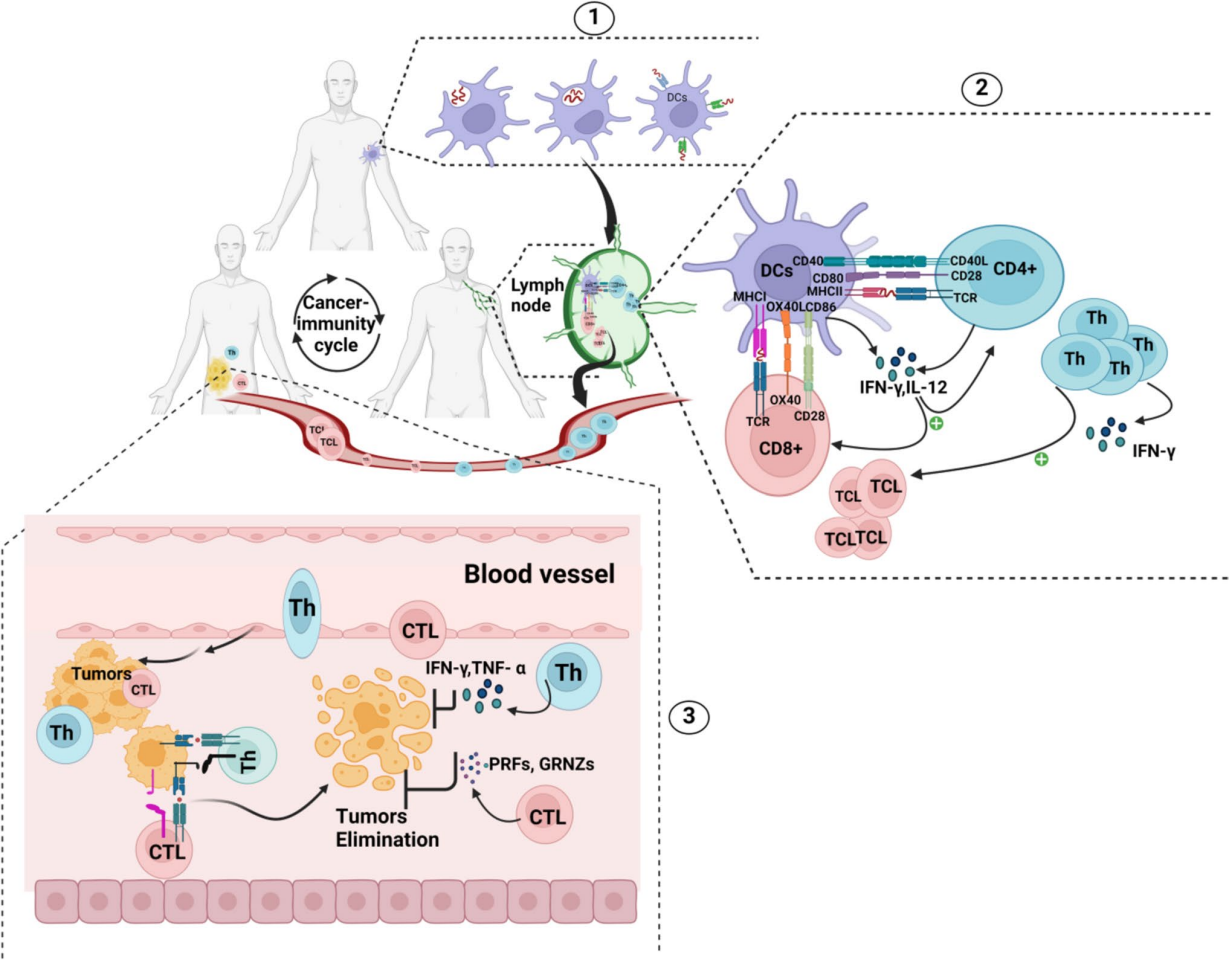
Principles of cancer-immunity interaction. 1: DCs encounter cancer antigens resulting from cancer cell death or from different cancer vaccine platforms, and after DCs internalize cancer antigens, they process them and load them onto MHC (I, II) on their surface membrane. 2: Once DCs have loaded tumour antigens, they migrate to lymph nodes via lymphatic vessels, where they present cancer antigens to lymphocytes via MHC II to CD4 + and MHC I to CD8 + . In addition to costimulatory molecules, they lead to T-cell priming and activation. After T cells expand and differentiate into effector T cells (Th and CTLs), they travel through blood vessels to infiltrate tumour sites; they bind tumour antigens exposed to cancerous cells via MHC molecules and kill them either by releasing inflammatory cytokines (IFN-γ and TNF-α) or inducing cancer cell apoptosis via their cytotoxic molecules (PRFs and GRNZs). Cancer epitopes can be captured by DCs peripherally or intratumourally, allowing the cycle to restart with refreshed and strengthened immune responses. CD40, CD80, CD86, CD40L, and CD28 are clusters of differentiation molecules located on the cell surface; they act as amplifiers of T-cell activation, differentiation, and influencers of their fate. IFN-γ and TNF-α are inflammatory cytokines implicated in several signalling cascades leading to pathogens elimination

In recent decades, several immuno-oncology studies have investigated the relationship between immune cells and cancer, revealing the mechanisms by which immune cells recognize tumour antigens and subsequently mount effector and memory immune responses. Numerous studies have shown that an immune-cancer response consists of multiple stepwise events to ensure the elimination of cancerous cells; this process is termed the cancer-immunity cycle, which was initially introduced in 2013 [20]. Briefly, this feedback loop starts when antigen-presenting cells (APCs) or dendritic cells (DCs) encounter cancer antigens; they upregulate their toll-like receptors (TLRs) to take up these circulating cancer antigens by phagocytosis and micropinocytosis and process them intracellularly, migrate to the lymph nodes and present them to T-cell lymphocytes [21]. DCs present cancer antigens to CD4 + T cells via MHC class II and to CD8 + T cells via MHC class I via costimulatory molecules and positive signals such as TCR, CD28, CD80/CD86, CD40, CD40L, OX40, OX40L, IL12, IFN-α, IFN-α and others [22–25]. The MHC-peptide-TCR complex with costimulatory factors results in the priming and activation of T cells. Once activated, T cells travel through blood vessels to infiltrate tumour sites and execute their effector functions. CD4 + T cells release several proinflammatory cytokines via their subpopulation of T helper (Th) cells to kill tumours; these cells also promote the expansion of CD8 + T cells and increase their function [26]. CD8 + T cells, after being activated and differentiated into cytotoxic T lymphocytes (CTLs), eliminate tumours by releasing different inflammatory cytokines (TNF-α and IFN-γ). Additionally, CTLs can promote the apoptosis of cancerous cells via the use of the cytotoxic molecules perforin (PRFNs) and granzymes (GRNZs) [27]. DCs also exhibit a tolerogenic function, engaging in the enhancement of central and peripheral tolerance. Therefore, DCs control effector and regulatory mechanisms relevant to the pathology of cancer and autoimmune disorders [28]. In addition, DCs and Th cells promote the activation of B cells to generate activated B plasma cells, the latter of which participate in tumour cell growth regression via antibody-dependent cellular toxicity (ADCC) or complement cytotoxicity activity [29] **(**Fig. 1**)**. It is important to note that this cycle normally leads to tumour growth inhibition. However, this is not the case in most cases due to the interference of many inhibitory factors in every step of the process and the resulting negative impact on the immune system components’ active reaction. In this context, cancer vaccines are one of the biological weapons used to circumvent the immunosuppressive role of this inhibitor cocktail and reunleash the cancer-immunity cycle.

## Mechanisms underlying tumour resistance to cancer immunotherapeutics

Although immunotherapeutics have revolutionized the way practitioners manage cancer disease treatments, several reports have demonstrated that patients respond differently to these treatments, with some patients showing low remission rates followed by relapse episodes (acquired resistance). In contrast, others fail to respond at the onset of treatment (primary resistance) [30–33]. Further investigations of these findings revealed that different inhibitory factors sometimes curb the strong immune response resulting from these treatments, i.e. intrinsic and extrinsic factors [34].

### Intrinsic factors

During disease, cancer cells rebel and adopt a multitude of strategies for resisting the destructive weapons of the immune system. One of the camouflaged ways that tumours take to escape the immune system is to alter or reduce T-antigen expression and presentation on their cell surface by downregulating MHC molecules, thus decreasing the immunogenicity and unrecognition of T lymphocytes [35]. Disruptions in IFN-γ signalling pathways reduce tumour cell sensitivity to T-cell-mediated destruction. This phenomenon can be attributed to mutational and transcriptional alterations in the IFN-γ signalling cascade (IFN-γ R/JAK/STAT), which is critical for the effector functions of IFN-γ. Such alterations lead to IFN-γ resistance and provide survival advantages to tumours. Furthermore, epigenetic changes at specific interferon-stimulated genes (ISGs) may result in their downregulation, further contributing to this resistance [36]. Moreover, tumour cells may generate and express immune checkpoint molecules (PDL-1) and immunosuppressive mediators, resulting in T-cell exhaustion [29]. In addition, cancer cells may undergo structural and morphological changes, transitioning from epithelial to mesenchymal phenotypes (epithelial to mesenchymal transition, EMT) and consequently increasing plasticity and mobility to create and colonize areas with low immune cell populations and densities (immune deserts) [37, 38].

### Extrinsic factors

**Fig. 2:**
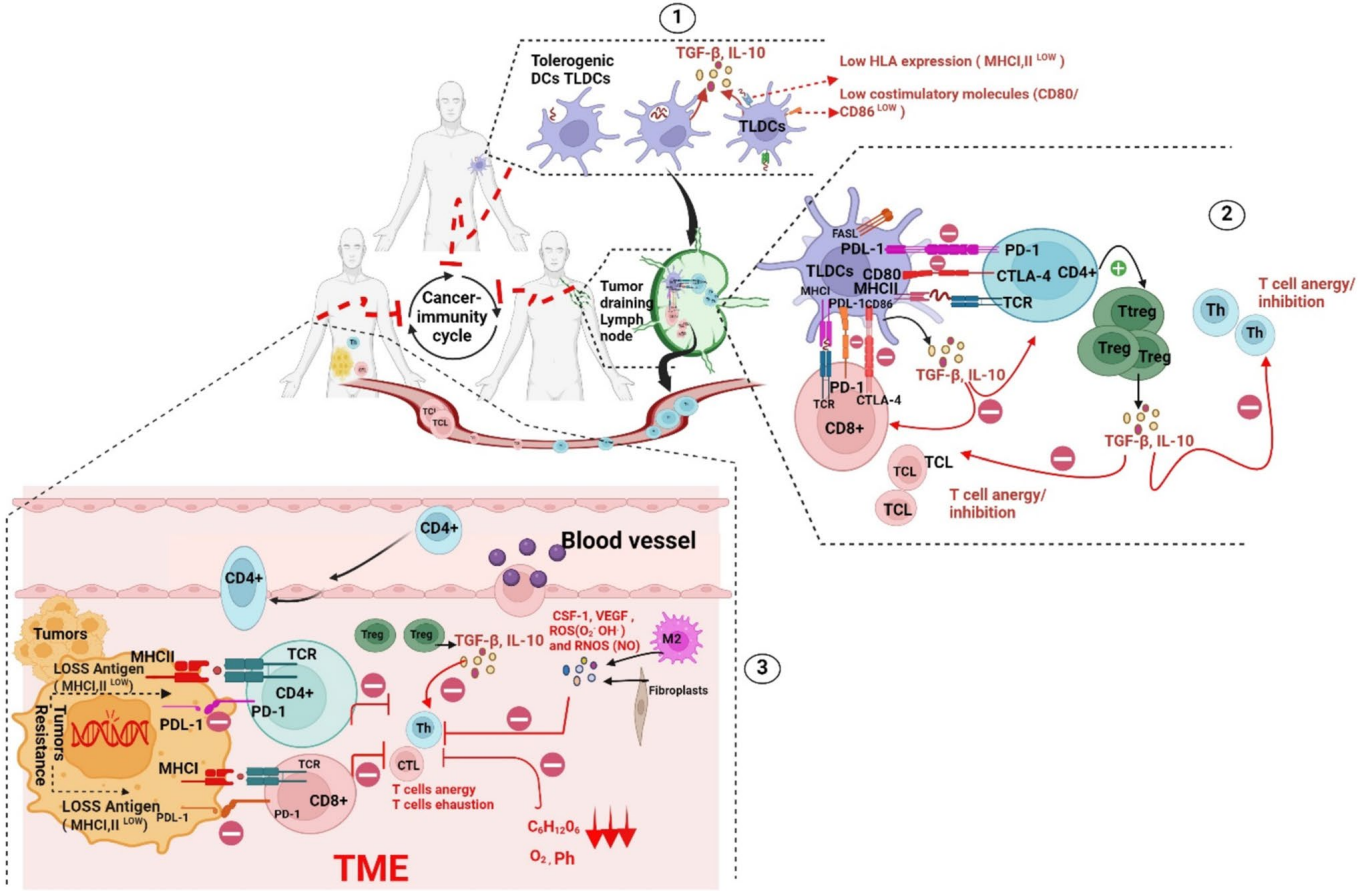
Examples of tumour resistance mechanisms. 1: Tolerogenic dendritic cells (TLDCs) are characterized by weak capture of cancer antigens, low expression of major histocompatibility complex (MHC) and costimulatory molecules, high expression of inhibitory molecules and regulatory cytokines (FAS, PDL-1, TGF-β, and IL-10) and impaired ability to promote T-cell activation and expansion. 2: After TLDCs migrate to the tumour-draining lymph node (TDLN), they bind to T cells via inhibitory molecules, resulting in T-cell deactivation. Moreover, TLDCs induce T-cell anergy by favouring Treg phenotype expansion. 3: Within the TME, cancer cells use their setups to escape the immune system, reducing cancer antigen exposure and altering some immune stimulatory signalling pathways, thus becoming unrecognized and insensitive to T-cell destruction. In addition, the metabolic stress conditions (low glucose levels, hypoxia, acidity, and oxidative stress) of the TME have a detrimental effect on T-cell functions, as they drive them towards exhaustion

Many studies have demonstrated the negative role of immune cells in inducing tumour growth and progression. Immunosuppressive tumours are characterized by multiple events in which innate and/or adaptive cells confer resistance to immune responses to tumours [39]. It has been shown that macrophages and natural killer (NK) cells can mediate tumourigenesis by creating an inflammatory niche via the release of proinflammatory cytokines [39]. Some chronic inflammatory episodes potentiate the risk of cancer malignancy initiation and installation, such as colon cancer, which in some scenarios could be preceded by inflammatory bowel disease (IBD) or *Helicobacter pylori* infections [40]. Moreover, macrophages can release molecules such as growth factors (VEGF, EGF and CSF-1), contributing to tumour severity and aggressiveness [41, 42]. On the other hand, CD4 + and CD8 + T cells trigger tumour escape by expressing immune checkpoint molecules (PD-1, LAG-3, CTLA-4, TIM-3) that bind to their related ligands on DCs and/or tumours, thereby stopping an immune reaction [43]. Additionally, Treg cells promote tumour resistance and restrict immune responses via their blocking mediators and cytokines (TGF-β, IL-10, and IL-35) [44]. Together, all these released molecules result in a highly immunosuppressive TME **(**Fig. 2**).**

## Cancer antigens

Despite all efforts to identify and determine the ultimate method of priming immune responses against cancer cells, designing antigen-based cancer vaccines still faces several challenges regarding i) the selection of antigens with optimal affinity for immune system cells, particularly T cells, and ii) their ability to elicit immune responses within the body and bypass the highly immunosuppressive TME. Tumour cells express different antigens that can be categorized as tumour-associated antigens (TAAs) or tumour-specific antigens (TSAs) [45]. TAAs might be overexpressed on cancerous cells and normal cells. Because TAAs are self-antigens, many populations of T cells that recognize them are eliminated during negative selection at the primary and secondary lymphoid organs [46]. Therefore, developing cancer vaccines targeting this type of antigen is challenging in terms of monitoring tolerance and inciting potent T-cell reactions. Conversely, TSAs are distinctly expressed in cancer cells, making them suitable targets for cancer vaccine development [47, 48]. TSAs regroup oncoviral antigens derived from viruses that cause cancer and neoantigens resulting from mutations in tumour cells [49, 50]. Several clinical studies have shown promising results in using vaccines targeting oncoviral antigens, reporting that using bivalent HPV vaccines targeting HPV 16/18-associated cancer was safe and efficacious in preventing invasive cervical cancer [51]. Other clinical trials have also demonstrated that the use of 4- and 9-valent HPV vaccines is highly immunogenic and well tolerated by trial participants [52]. Moreover, other clinical studies using HBV vaccines have shown interesting outcomes in preventing the occurrence of virus-mediated liver malignancies [53]. It is important to emphasise that the several years of clinical trials using traditional prophylactic vaccines have enabled the scientific community to gain significant mechanistic insights into immune responses to vaccines. Accordingly, investigators have progressed to another level of scientific reasoning by formulating more accurate vaccines targeting T-cell responses rather than limiting them to B cells only. Such efforts have opened new perspectives in cancer vaccine development [54].

## Cancer vaccine platforms

The basic principle behind all cancer vaccines is to target-specific cancer antigens [48] to mobilize the host’s immune system components to eradicate cancer cells through a series of stepwise events of the cancer-immunity cycle. Many cancer vaccine platforms have been developed to achieve this goal. From a clinical point of view, the preferred platforms are those that offer more benefits than drawbacks in terms of safety, potency, and persistence of immune reactions. In the following chapters, different cancer vaccine strategies will be described.

### Peptide-based cancer vaccines

**Fig. 3:**
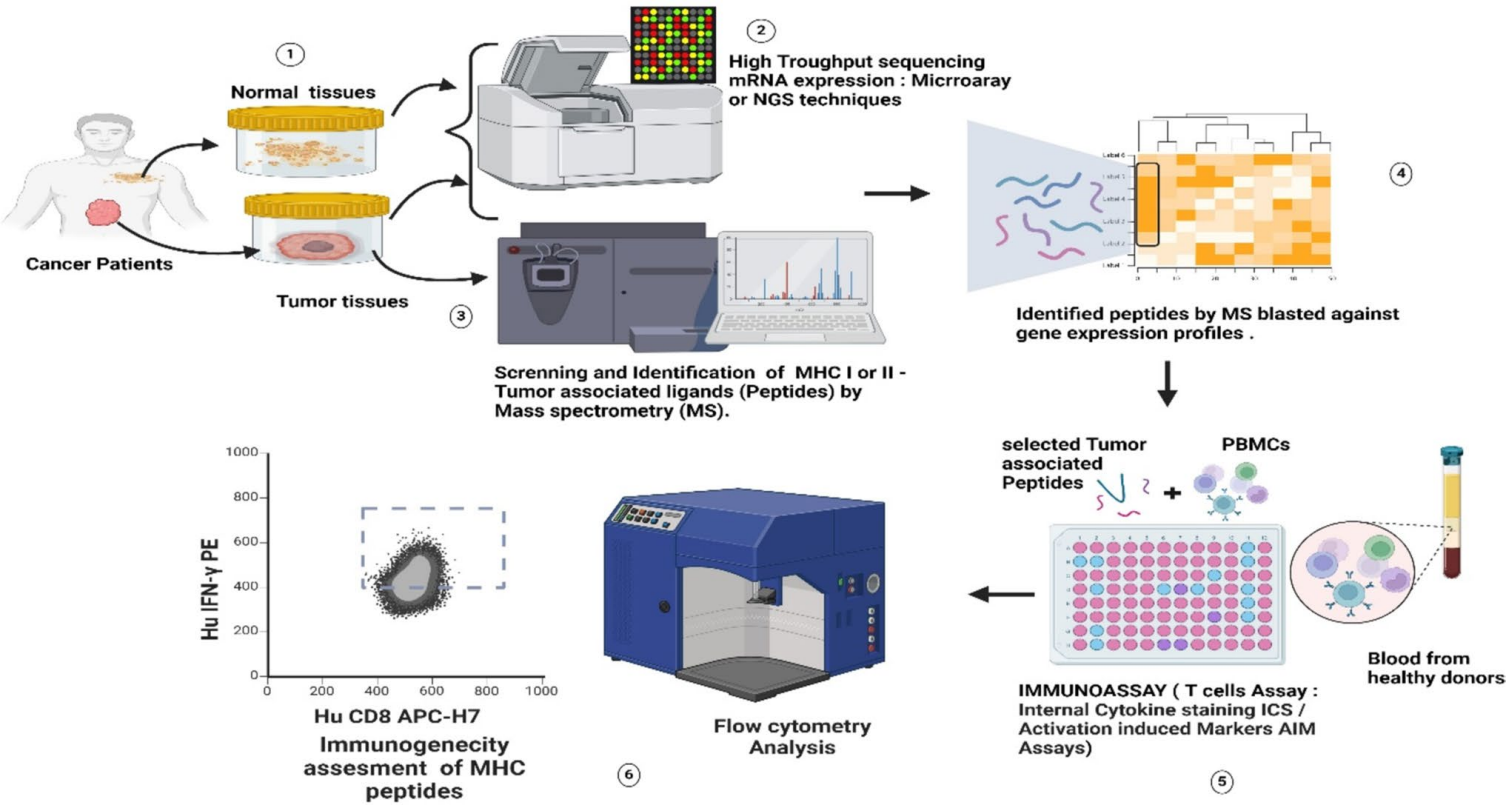
Tumour-associated peptide-based cancer vaccines: the diagram briefly illustrates methods used to select and design personalized tumour peptide-based cancer vaccines. **1** Tumour and normal tissues were harvested from cancerous patients. **2** Comparative transcriptomic analyses were performed to evaluate mRNA transcript expression in both tissues using microarray or NGS techniques. **3** Tumour tissues are also used to characterize and identify MHC peptides by mass spectrometry (MS). Four peptide sequences were identified using search engines to map the acquired spectra on either general protein databases (such as UniProt) or custom proteome databases originating from the transcriptome assembly (proteogenomic approach). **5** A handful of peptides are selected and tested for their immunogenicity by incubating them with peripheral immune cells (PBMCs) from healthy donors.** 6** T-cell reactivity towards the screened tumour-associated MHC peptides was assessed by an ICS using flow cytometry, and only highly immunogenic peptides were chosen as the best candidates for vaccine formulations

The key feature of tumour-derived peptide-based therapeutics is their immunogenic properties. Predicting the antigenicity of peptides is not straightforward [22, 23]. This depends on several factors related to the structure, size, polarity and hydrophobicity of the amino acid residues forming these peptides. However, despite these hurdles, taking advantage of high-throughput experiments [55, 56], many tumour-associated peptides have been successfully discovered and used as drug substances for personalized peptide-based cancer vaccine formulations (Fig. 3**)**. Upon injection, short peptides (8–10 amino acids) bind to their related MHC molecules and incite T-cell responses [57]. As T cells recognize specific parts of antigens (epitopes), MHC-tumour-derived peptide complexes often carry a small portion of amino acids corresponding to T-cell epitopes and thus enhance their affinity profile [58]. CD4 + Th cells are involved in CD8 + TCL activation and promotion. Long MHC II peptides (approximately 30 amino acids) have gained considerable attention in the drug discovery process; these long MHC II peptides, once injected, are captured by DCs and presented to CD4 + cells, which are activated and selectively kill cancer cells [59, 60]. Clinical trials are ongoing to determine the safety and efficacy of peptide-based cancer vaccines **(**Table 1**).**

**Table 1:** Recent clinical trials of peptide-based cancer vaccines

Clinical Trial ID	Target (peptide)	Indication	Phase	Study completion date
NCT03559413	Patient-individualized peptide	Acute Lymphoblastic Leukaemia	1, 2	2023
NCT04688385	Multipeptide vaccine	Leukaemia	1	2024
NCT05475106	Neoantigen peptides	Different types of cancer	1	2024
NCT04842513	Multipeptide	Glioblastoma	1	2025
NCT04270149	ESR1 peptide vaccine	Breast cancer	1	2026

### Nucleic acid-based cancer vaccines:

The use of nucleic acids (DNA and RNA) is another interesting approach for formulating effective cancer vaccines. Conceptually, DNA vaccines are more cost effective, easier to manufacture, target-specific and safer than other platforms [61]. DNA sequences for cancer vaccines are engineered to express and encode different types of tumour antigens, thereby inciting immune cells to kill diseased cells expressing such antigens [62]. DNA constructs can be delivered directly as naked fragments or encapsulated in different vectors. When they are administered into the body, they enter cells to undergo transcription in the nucleus and translation in the cytosol. After being translated into antigens of interest, they are presented to T cells by competent antigen-presenting cells, triggering cancer-immunity cycle events [63]. DNA constructs have additional advantages; these costly fragments can be fused to other sequences expressing costimulatory and immunomodulatory molecules, which enhance their ability to mount anticancer immune responses [64–67]. Many preclinical and clinical studies have proven the therapeutic effect of DNA-based cancer vaccines; in this regard, preclinical studies using tumour-bearing mice have shown that DNA vaccines targeting fibroblast activation protein (FAP) provide potent antitumour immune responses in these rodents [68]. A plasmid DNA vaccine targeting the G250 gene strongly delayed tumour growth in preclinical models [69]. Additionally, the VGX-3100 DNA plasmid targeting the E6 and E7 proteins of the HPV-16 and HPV-18 viruses has demonstrated potent immunogenicity and conferred significant protection for patients with grade 2 and 3 cervical intraepithelial neoplasia (CIN2/3) [70]. Furthermore, the GX-188E DNA vaccine encoding the E6 and E7 oncogenes effectively protected patients with cervical intraepithelial neoplasia 3 [54]. Nucleic acid vaccines have low immunogenicity, and large quantities of DNA are needed to achieve strong immune responses [71], possibly triggering autoimmune diseases as a side effect [72–74]. These limitations are slightly limiting the balance in favour of mRNA-based cancer vaccines. Although mRNA constructs face issues of stability and enzymatic degradation [75], they do not integrate into the host genome for translation and hence are free of related oncogenic risks, in contrast to DNA vaccines [75, 76].

Similar to DNA constructs, mRNA motifs are designed to express antigens and elicit immune responses [77]; they can be administered as naked molecules or as carriers. Furthermore, they are processed faster than DNA motifs [76, 78]. During covid pandemic, mRNA vaccines targeting coronavirus antigens have played a significant role in preventing millions of deaths and contributing to the end of the COVID-19 global health emergency; to that, scientists behind mRNA COVID vaccines were awarded the NOBEL prize of Medicine in 2023 [79, 80]. Upon injection, they undergo a series of stepwise events within the cancer-immunity cycle, resulting in anticancer efficacy [81]. Some clinical trials using mRNA-based cancer vaccines have demonstrated promising results. A phase I study using an RNA-LPX vaccine targeting four tumour antigens revealed potent CD4 + and CD8 + immune responses with tumour regression in patients with unresectable melanoma [82]. Additionally, a phase I clinical trial testing a personalized RNA neoantigen vaccine in patients with pancreatic ductal adenocarcinoma (PDAC) revealed sustained T-cell immune responses with delayed tumour recurrence in those patients in the same context. Follow-up phase II of this study is ongoing [83], and many other clinical studies are underway **(**Table 2**)**.

**Table 2:** Selected clinical trials of mRNA-based cancer vaccines

Clinical trial ID	Vaccine type	Indication	Phase	Completion date
NCT03897881	mRNA-4157	High-risk melanoma	2	2029
NCT05142189	mRNA vaccine-BNT116	Advanced non-small cell lung cancer	1	2027
NCT06195384	Neoantigen mRNA vaccine	Solid tumours	1	2037
NCT06326736	Neoantigen mRNA vaccine SJ-Neo006	Resectable pancreatic cancer	1	2026
NCT04534205	mRNA vaccine-BNT113	Metastatic Head and Neck Cancer	2	2028

### Viral vector-based cancer vaccines

Historically, viruses have been used as attenuated or inactivated vaccines to treat and prevent infectious diseases [84]. Due to their natural immunogenic properties, viruses are used as effective carriers of tumour antigens. Many viruses are genetically engineered to safely deliver targeted antigens and elicit potent and long-lasting immune responses [85, 86] (Table 3**)**.

**Table 3:** Recent ongoing clinical trials based on vector cancer vaccines

Clinical trial ID	Vector	Indication	Phase	Study completion date
NCT05419011	Multitargeted recombinant adenovirus 5	Colon and other cancers	IIb	2027
NCT04695327	TNFα and IL-2 coding oncolytic adenovirus TILT-123	Advanced solid tumour	I	2025
NCT02705196	LOAd703 oncolytic virus	Pancreatic cancer	I/II	2025
NCT04041310	Nous-209 (Adenovirus GAd20-209-FSP + modified vaccinia virus ankara MVA-209-FSP)	Microsatellite unstable solid tumours	I/II	2026
NCT04990479	Nous-pev (GAd-PEV + MVA-PEV)	Lung cancer and melanoma	I	2024
NCT04908111	ChAdOx1-MAGEA3-NYESO	Non-small cell lung cancer (NSCLC)	I	2025
NCT02285816	AdMA3	Advanced/metastatic solid tumours	II	2024
NCT05812677	Oncolytic herpes simplex virus type I (R130)	Cervical and endometrial cancer	I	2026
NCT02779855	Oncolytic modified herpes simplex 1 virus	Invasive breast carcinoma	I/II	2024
NCT05914376	Recombinant human IL-21 oncolytic vaccinia virus	Advanced solid tumours	I	2025
NCT05788926	Oncolytic vaccinia virus encoding IL-12 and anti-CTLA4	Metastatic non-small cell lung cancer	I	2025
NCT04215146	Oncolytic reovirus	Breast cancer metastatic	II	2024
NCT01961063	Lentiviral vector rHIV7-shI-TAR-CCR5RZ transduced hematopoietic progenitor cells	AIDS-related non-Hodgkin lymphoma	I	2024
NCT02483312	Lentivirus engineered to express IL-12	Acute myelogenous leukemia (AML)	I	2024
NCT05492682	Replicating adenovirus-1 (PeptiCRAd-1)	Melanoma (Skin)	I	2025
		Triple-negative breast cancer		
		Non-small		
		Cell lung cancer		
		Synovial sarcoma		
		Myxoid Liposarcoma		
		Colorectal cancer		
		Triple-negative breast cancer		
		Non-small		
NCT02496273	CEA specific AAV	Gastric cancer	I	2030

#### Adenoviruses as viral vectors

**Fig. 4:**
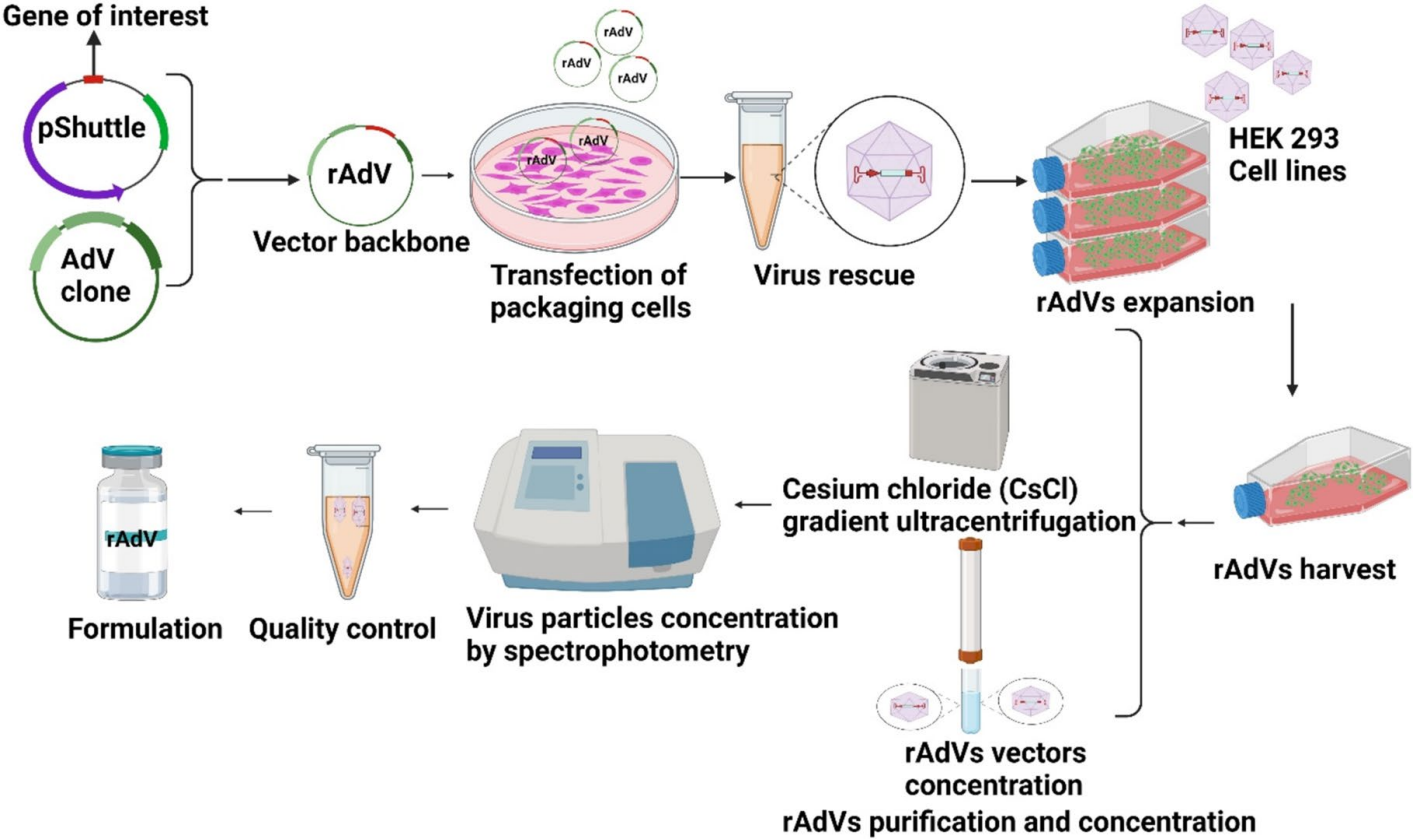
Laboratory-scale production of adenovirus vector-based cancer vaccines [119] Based on the desired clinical benefit, recombinant adenovirus vectors (rAdVs) designed with genes of interest, including tumour-associated antigens, were cloned and inserted into shuttle plasmids. Subsequently, genes are integrated into the adenovirus genome. The adenoviral backbone carrying genes of interest might delete viral replication regions (E1 or E3 codon regions) in the case of replication-defective AdV vectors or modify the viral genome to generate adenovirus replicative-competent vectors that replicate exclusively in cancer cells. Once the rAdV backbone vector is engineered, packaging cells (e.g. the HEK 293, A549, and PERC6 cell lines) are transfected with linearized rAdV clones. After the cytopathic effect (CPE) occurred, the adenovirus vectors were restored, the cells were allowed to proliferate, and the cells were harvested when the viral plaques became visible under a microscope. Harvested rAdV vectors were purified by caesium chloride gradient ultracentrifugation and concentrated through flow columns. Their concentration was calculated by spectrophotometry and calculated as the number of virus particles (Vp)/ml. Before formulation, rAdVs undergo quality control testing to confirm their genetic stability and assess their potency and immunogenicity. Production methods can be optimized and scaled up to meet industrial manufacturing process requirements

Adenoviruses (AdVs) used for clinical applications are mainly represented by serotypes 5 and 2 (AdV5 and AdV2) [87–89]. Structurally, AdVs are nonenveloped viruses with linear double-stranded DNA genomes ca. 40 kb in size [90]. Targeted genetic modifications of viral genomic regions in these adenovirus (AdV) generations have enabled the development of recombinant adenoviral vectors (rAdVs). These vectors offer a high gene insertion capacity (approximately 8 kb), enhanced tropism, improved stability, and reduced toxicity [91–94] (Fig. 4**)**. rAdVs are used either as replication-deficient adenovirus vectors to deliver and express tumour antigens or as oncolytic replication-competent AdVs, which selectively replicate within and lyse cancer cells [94–101]. AdV vectors offer many advantages over other platforms, including short manufacturing times [102], favourable safety profiles and characterization [103], and no integration into the host genome [104]. Moreover, they can infect both dividing and nondividing cells, thus targeting a wide range of cell types [105, 106]. Many preclinical studies have demonstrated their promising therapeutic application for eradicating cancer cells. In this context, treatment with AdV3 expressing human CD40L (Ad3-hTERT-CMV-hCD40L) completely improved the survival rate (100%) in a treated humanized mouse model compared to that in controls and resulted in potent antitumour-specific Th1 immune responses [107]. Moreover, using the [108] adenovirus ONCOS-102 (AdV5/3-D24-GM-CSF) in a mesothelioma-bearing mouse model elicited strong antitumour T-cell responses [109]. At the clinical level, patients with treatment-refractory and immune cell-poor solid tumours treated with ONCOS-102 showed satisfactory antitumour CD8 + T-cell responses [110, 111]. In addition, an interesting recent study [100] revealed that adenovirus armed with tumour necrosis factor alpha and interleukin-2 (TILT-123-igrelimogene litadenorepvec) was safe and produced antitumour effects in patients with advanced solid cancers [112]. To date, two viral vector-based cancer vaccines have been approved by the US FDA. One of them, Nadofaragene firadenovec (nadofaragene firadenovec-vncg; Adstiladrin®), developed by Ferring Pharmaceuticals, obtained its first global approval in the USA in December 2022. Nadofaragene firadenovec is a nonreplicating adenoviral vector-based cancer vaccine encoding (IFN)-α2b that is used to treat patients at high risk of Bacillus Calmette-Guérin (BCG)-unresponsive nonmuscle invasive bladder cancer (NMIBC). Despite promising clinical results, more efficacious treatments are needed. Therefore, more efforts are being made to increase the efficiency of adenovectors in priming T-cell responses and developing novel platforms. One such approach [113] involves coating adenovirus vectors with MHC I tumour peptides, termed peptide-coated conditionally replicating adenovirus (PeptiCRAd). Preclinical studies have demonstrated the T-cell priming efficacy and antitumour potency of these compounds in melanoma, mesothelioma and colon-bearing mouse models [114–118]; thus, promising data have encouraged investigators to move forward to clinical trials (Table 3**)**.

**5.3.2**- **Adeno-associated viruses as viral vectors.**

**Fig. 5:**
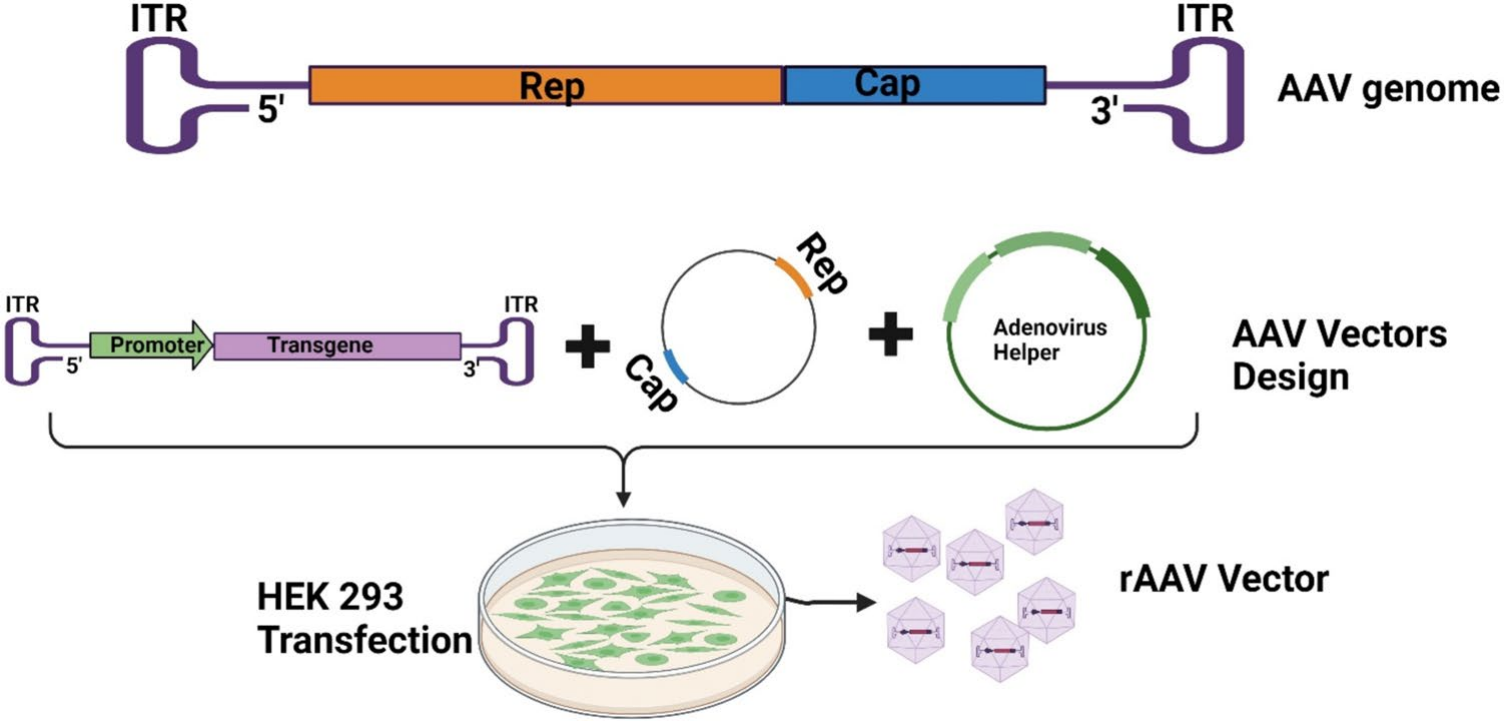
Design of recombinant adeno-associated virus (rAAV) vectors. Once the rAAV backbones are constructed, packaging HEK293 cells are transfected to expand the rAAV vectors. After expansion, the rAAV vectors were purified, and their concentration was calculated by spectrophotometry

AAVs were first discovered in 1965 as contaminants of adenovirus preparations [116, 120]. Later, AAVs were identified as viruses that need the presence of adenovirus to replicate; otherwise, they integrate into the host genome, specifically on chromosome 19 [121]. AAVs are nonenveloped viruses of the parvovirus family. Their genome consists of single-stranded DNA (ssDNA) that is 4.7 kb in length and flanked on both ends by inverted terminal repeats (ITRs). The genome of AAVs consists of two coding regions for replication (Rep) and encapsidation (Cap). These regions are often deleted and replaced by an expression cassette harbouring genes of interest [122, 123] (Fig. 5**).** Recombinant AAVs (rAAVs) share the same advantages as AdVs, except for their packaging capacity, which is significantly lower (less than 4.4 kb) than that of AdVs [124–126]. Multiple preclinical studies have tested the efficacy of AAV vector-based cancer vaccines. Using a rAAV6 vector expressing tumour antigens suppressed tumour progression and elicited humoral and cellular tumour antigen-specific responses in melanoma-bearing mouse models [127]. Furthermore, treating a carcinoembryonic antigen (CEA) transgenic mouse model with rAAV expressing CEA was efficacious and provided a potent antigen-specific antitumour response [128]. To date, no rAAV cancer vaccine candidates have been approved, and only one ongoing clinical trial is currently investigating this type of vaccine (Table 3**)**.

#### Herpes viruses as viral vectors

Herpes simplex viruses (HSVs) are another interesting platform for developing cancer vaccines. HSVs belong to the Herpesviridae family. Their genome consists of double-stranded DNA (dsDNA) with a size within the range of 70 to 240 Kb. HSV type 1 (HSV‐1) is the most widely used HSV in medical applications [129, 130]. HSV-1 vectors can insert up to 50 Kb of a foreign DNA sequence without incorporating the host genome for replication [131]. Interestingly, herpes viruses have a natural tropism for neuronal cells, making them suitable vectors for gliomas and glioblastomas [132–135]. Like AdV vectors, HSV vectors are used either as replicative deficient HSV vectors to deliver and express genes of interest or as oncolytic vectors that are replication competent to selectively replicate in and lyse cancer cells [136]. Many preclinical studies have shown the therapeutic value of HSV vector-based cancer vaccines in mouse models. In this regard, one recent study demonstrated that intratumour infiltration of activated T cells was accompanied by tumour metastasis inhibition in a breast cancer mouse model treated with an oncolytic HSV-1 VC2-vectored vaccine [137]. Talimogene Laherparepvec (T-VEC) is a modified oncolytic herpes simplex virus-1-based cancer vaccine commercialized by Amgen. It was the first oncolytic virus to be approved by the FDA and EMA for treating patients with unresectable metastatic stage IIIB/C–IVM1a melanoma [138–141]. Other HSV-based cancer vaccines have also been tested in clinical trials. RP1, which is a replication competent, enhanced-potency oncolytic HSV-1, has demonstrated durable antitumour activity in patients with skin cancers [142], while OH2, an oncolytic herpes simplex virus type 2 engineered to express GM-CSF, was tolerable and demonstrated potent antitumour immunity in patients with metastatic esophageal and rectal cancer (Table 2) [143].

#### Vaccinia virus as a viral vector

Vaccinia virus (VV) was first used to treat and prevent smallpox [144–146]. VV is an enveloped virus of the Poxviridae family with a dsDNA genome of approximately 190 kb in length [147]. Many advantages are attributed to VV. It can accommodate up to 40 Kb of foreign gene inserts and has high transduction efficiency, stable antigen expression and a nonintegrating genome [147–149]. VV can be used as a carrier to deliver genes of interest. For this purpose, VV strains are attenuated by several passages to render them replication defective; VV can also serve as an oncolytic vector to kill tumour cells by deleting or modifying the thymidine kinase (TK) locus of the VV genome, thus creating replication-competent VV strains [147, 150]. Several preclinical and clinical oncology studies have investigated the therapeutic effect of VV-vectored vaccines. In one study, the oncolytic Vaccinia strain Guang9 (VG9) conferred significant antitumour effects and potent cytotoxic T‑lymphocyte responses in a murine melanoma tumour model [151]. In another study (NCT00554372), the oncolytic immunotherapeutic vaccine JX-594 significantly improved overall survival in patients with advanced carcinoma [152]. Moreover, treating patients with advanced head and neck carcinoma with oncolytic vaccinia virus (GL-ONC1) has improved overall survival with satisfactory safety [153]. Other recent clinical trials are underway (Table 2**).** Like AdV and HSV vectors, VV-vectored vaccines are confronting some hurdles related to immunogenicity and safety. Therefore, massive effort is directed towards making them more effective.

#### Reovirus and lentiviral vectors

Lentiviruses (LVs) are Retroviridae viruses. These viruses are enveloped and nonreplicating viruses carrying a single-stranded (ss) RNA of approximately 8 kb in length [135]. LVs can package 9 kb for gene insertion; they can infect both dividing and nondividing cells and exhibit stable transgene expression. Nevertheless, they integrate into the host genome, which carries the risk of oncogenesis [154, 155]. Most LVs that are used to deliver genes are derived from human immunodeficiency virus (HIV) type 1 [156]. They serve as a potent vector to generate engineered cell-based cancer therapies [157].

Reoviruses (Reos) belong to the Reoviridae family. These viruses are enveloped viruses with genomes containing 11 kb of double‐stranded (ds) RNA. Compared to LVs, Reo can infect only dividing cells and integrate into the host genome. For this reason, similar to LVs, they raise concerns regarding their oncogenic potential [147, 150, 158, 159]. Many reports have demonstrated the efficacy of Reo vector-based cancer vaccines; in one preclinical study, a reovirus-based agent induced endoplasmic reticulum stress-mediated apoptosis in pancreatic cancer mouse models [160]. Furthermore, REOLYSIN, a live replication-competent Reovirus Type 3 Dearing strain, was efficacious and triggered a strong antitumour immune response in patients with advanced malignant melanoma [161]. Based on these findings, the FDA has granted an orphan drug designation (ODD) to Reolysin for the treatment of pancreatic cancer and malignant gliomas [162].

### Bacillus Calmette-Guérin (BCG) vaccine

For more than three decades, Bacillus Calmette–Guérin (BCG) has been the gold standard treatment for nonmuscle invasive bladder cancer (NMIBC) [163]. Despite its long-established use, there is still ambiguity regarding the mode of action of this biotherapy once applied by intravesical instillations [163, 164]. The administered BCG particles are internalized by bladder cancer cells and DCs and presented to immune cells, which results in anti-bladder tumour immunity mediated by cytotoxic CD8 + T cells, NK cells, macrophages and TRAIL granulocytes [165, 166]. Despite the great therapeutic value of the BCG vaccine, severe adverse effects may occur, with more than 70% of patients experiencing some form of toxicity [167], urinary frequency, cystitis, fever and hematuria [168]. As with other vaccines, a few limitations are linked to the BCG vaccine; approximately one-third of NMIBC patients are nonresponders, while others face relapse episodes [169]. There are also other logistical and technical problems, such as global BCG shortages and efficacy profile issues, among different BCG substrains [170, 171]. Many studies have been carried out to identify alternative treatments for nonresponding BCG patients. As mentioned above, Nadofaragene firadenovec was approved for treating these patients. Certain improvements have also been made in the BCG production process, and new treatment guidelines have been adopted to address BCG shortage concerns [172, 173].

### Cell-based cancer vaccines

Engineering patients’ own immune cells (DCs, T cells, and NK cells) is another interesting strategy for developing cancer vaccines, consisting of harnessing the immune system to eradicate cancer. Furthermore, various cellular vaccines have been designed using either whole tumour cells delivering antigens or cell lysates serving as a source of tumour antigens [174].

#### DC-based cancer vaccines

Given the ability of DCs to induce antitumour responses by taking up tumour antigens and priming T and B immune cells, leading to cancer-immunity cycle events, multiple DC-based cancer vaccines have been developed. These patients are currently undergoing clinical trials (Table 4**).** DCs can be used to design cancer vaccines in different ways. Autologous immature DCs can be harvested and isolated from peripheral blood monocytes or CD34 + hematopoietic stem cells (HSCs) [175–177]. Once harvested, these immature DCs are activated ex vivo to generate mature DCs. This could be achieved by their stimulation with a cytokine cocktail (IL-1β, IL-6, TNF-α or CD40L) [178–180] or with mRNAs encoding the proteins CD40L, CD70 and caTLR4 via electroporation [181]. Mature DCs can be reinfused into patients directly or after preloading with tumour antigens [182]. Only one DC-based cancer vaccine (sipuleucel-T (Provenge) developed by Dendreon Pharmaceuticals LLC) has been approved by the FDA -. Sipuleucel-T, an autologous ex vivo DC vaccine, was found to be safe and showed potent efficacy in patients with advanced prostate cancer [183, 184]. Despite this clinical success, DC-based cancer vaccines face some restrictions that limit their clinical efficacy. DCs originating from monocytes are frequently used in clinical trials [185]. However, the heterogeneity of DC subpopulations makes it difficult to predict which subset is the most effective [186]. Therefore, formulating vaccines with heterogeneous subsets can mimic in vivo conditions and seems to be more beneficial [187]. Moreover, the inefficient in vivo migration of DCs to tumour-draining lymph nodes is another factor affecting the efficacy of DC-based cancer vaccines [188]; thus, in situ administration strategies have been tested and shown to be promising [189].

**Table 4:** Recent ongoing clinical trials of DCs and tumour-based cancer vaccines

Clinical Trial ID	Target	Indication	Phase	Study completion date
NCT04348747	Anti-HER2/HER3 Dendritic Cell Vaccine	Brain Metastasis From Triple Negative Breast Cancer or HER2 + Breast Cancer	IIa	2026
NCT03546361	Autologous Dendritic Cell-AdenovirusCCL21 Vaccine	Stage IV Non-small Cell Lung Cancer	I	2025
NCT05809752	Dendritic Cell Vaccine Against HER2/HER3	Breast Cancer	I	2026
NCT05127824	Autologous alpha DC1/TBVA vaccine	Kidney Cancer	IIa	2026
NCT05317325	Autologous DCs pulsed with HOCl-oxidized autologous tumour lysate	Esophageal Squamous Cell Carcinoma	I	2024
NCT05773859	Autologous tumour lysate-loaded autologous XP-DC (cDC1)-based vaccine	Ovarian cancer	I/II	2024
NCT04239040	Autologous neuroblastoma cell vaccine (GVAX)	Neuroblastoma	I	2024
NCT05559177	Chimeric Exosomal Tumour Vaccines	Metastatic Bladder Cancer	I	2023
NCT06023277	ConvitVax autologous tumour cells	Metastatic Breast Cancer	I/II	2027
NCT05642195	Cancer Lysate Vaccine: H1299 Cell Lysates	Negative Non-Small Cell Lung Cancer	I/II	2035
NCT03807102	Tumour Vaccine	Lung Cancer	I/II	2026
NCT03395587	Autologous, tumour lysate-loaded, mature dendritic cells (DC)	Glioblastoma	II	2025

#### Tumour cell-based cancer vaccines

Unlike other cancer vaccine platforms that use a specific and narrow antigen repertoire, tumour cell-based cancer vaccines comprise a wide-ranging panel of tumour antigens, thereby providing an attractive alternative to design potent cancer vaccines offering strong and long-lasting tumour-specific immune responses [190, 191]. Tumour-based cancer vaccines can also be used as whole tumour cell lysates, which are prepared by irradiation with ultraviolet B or repeated freeze‒thaw processing [190]. Such manipulation of whole tumour cell lysates leads to the exposure of phosphatidylserine (PS) on the tumour cell surface and to the release of apoptotic mediators (high mobility group box 1-HMGB1, calreticulin-CRT and pentraxin-3-PTX3), thus enhancing DC stimulation and capture capacity and resulting in the reversal of cancer-immunity cycle events [190, 192–194]. Exosomes and RNAs derived from tumour cells constitute another source of tumour antigens used in tumour-based vaccine formulations [195–198]. Some tumour cell-based cancer vaccines have been tested clinically. GVAX is a whole tumour cell-based vaccine that is genetically modified to express and secrete GM-CSF. Autologous and allogeneic GVAX formulations are being tested in several cancer types, including pancreatic cancer, colorectal cancer, and neuroblastoma, either alone or in combination with other drugs. Clinical studies using GVAX have shown poor outcomes and limited efficacy in patients with pancreatic cancer [199, 200]. The GVAX® vaccine, which consists of two myeloma cell lines and K562 cells modified to express GM-CSF, is undergoing a phase II clinical trial in multiple myeloma patients **(NCT03376477)**. Melacine is another vaccine that consists of a lysate of two melanoma cell lines. Following an evaluation in a phase III study [201, 202], Canadian approval was granted for treating stage IV melanoma [203]. Other clinical trials using tumour-based cancer vaccines are underway (Table 4**)**.

## Cancer vaccines–hurdles and overcoming strategies

**Fig. 6:**
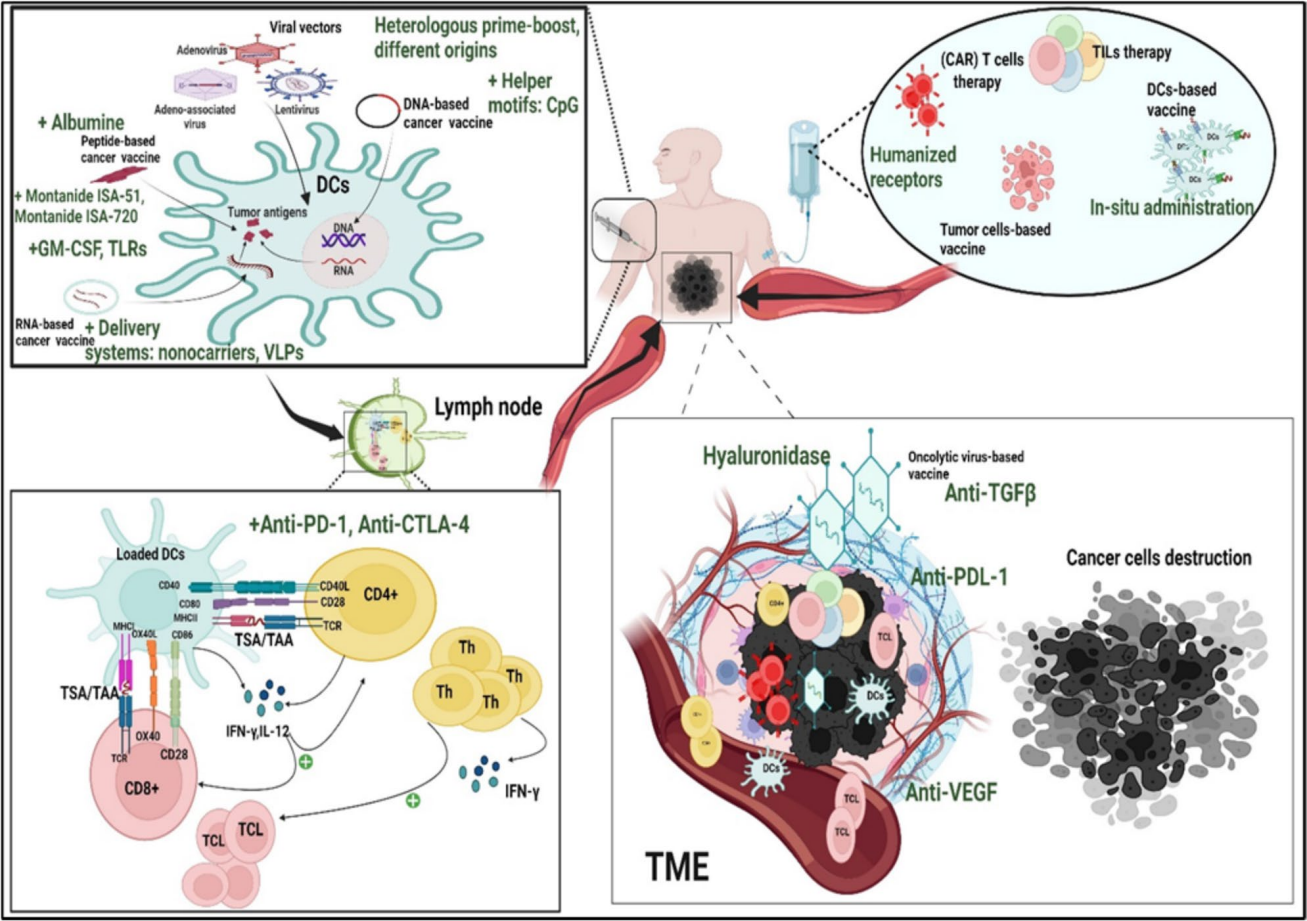
Mechanism of action and mitigation strategies for cancer vaccines. This diagram illustrates the mechanism of action of different cancer vaccines covered throughout this review. Diverse mitigation strategies (highlighted in green) are employed to enhance the efficacy of cancer vaccine platforms and unleash the cancer-immunity cycle

Despite the great therapeutic success of cancer vaccines proven by positive outcomes in numerous clinical trials, these immunotherapies still face some setbacks and hurdles that hinder their potential benefit in managing malignancies. As noted throughout this review, the design of cancer vaccines needs to be accompanied by the use of mitigation strategies to overcome major limitations related to the immunogenicity profile, targeted vaccine delivery, preexisting immunity and tumour resistance that significantly affect vaccine efficacy **(**Fig. 6**).**

### Immunogenicity and vaccine delivery

Diverse approaches have been employed to address the immunogenicity issues shared by virtually all vaccine platforms, though to varying degrees. The combination or direct conjugation of peptides with adjuvants such as Montanide ISA-51, Montanide ISA-720 and Detox or with immunostimulatory cytokines (IFN-α, IFN-γ, IL-2, IL-15, and GM-CSF) and immunomodulatory molecules (pattern recognition receptor-PRRs and Toll-Like receptor-TLR) potentiates the immunogenicity of peptide-based cancer vaccines, thus inducing APC activation and eliciting strong immune responses [204–209]. Moreover, conjugating peptides to albumin moieties increases their lymphatic trafficking and immunogenicity, resulting in DC activation and T-cell priming [210]. Similarly, arming DNA-based cancer vaccines with helper motifs such as unmethylated CpG dinucleotides or linking them to immunostimulatory cytokines or immunomodulatory fusion constructs has notably boosted the immunogenicity of these platforms [211–213]. In this context, many delivery systems have been developed to improve vaccine efficacy and persistence, with liposomes, virus-like particles (VLPs) and nanocarriers (NCs) serving as potent carriers for DNA, mRNA and peptide cancer vaccines, protecting them from degradation and ensuring delivery directly to competent cells (DCs), hence strengthening mounted immune responses [214–217].

### Preexisting immunity

Many reports have emphasised that adenovirus and adeno-associated virus vector-based cancer vaccines face major challenges related to preexisting immunity [218]. Since humans are naturally infected with adenoviruses, they mount immune responses against them [218, 219]. Therefore, the use of these vectored vaccines in patients triggers immune-mediated responses directed against viral capsids or transgene products. This is reflected by high rates of neutralizing antibody (nAb)- and AdV- or AAV-specific T-cell responses. Such events dampen vaccine efficacy and reduce vaccine immunogenicity [220, 221] using a myriad of technical solutions may address this concern. Some studies have shown that opting for a heterologous prime-boost option—delivering transgenes with different AdV serotypes in the prime and booster shots—may circumvent preexisting immunity. In clinical studies, this strategy has proven its efficacy in generating high levels of memory T cells. It has been demonstrated that the heterologous recombinant adenovirus (rAd)-based vaccine, Gam-COVID-Vac (Sputnik V), showed a good safety profile and induced strong humoral and cellular immune responses in participants in phase 1/2 clinical trials. This approach was effectively demonstrated in malaria vaccination, where a heterologous prime-boost regimen—using a plasmid DNA vaccine followed by a recombinant modified vaccinia virus Ankara (MVA)—successfully elicited high frequencies of antigen-specific, IFN-γ-secreting T-cell responses in humans [222–225]. In addition, adenovirus vectors derived from nonhuman primates (NHPs), such as chimpanzees, have a low seroprevalence of neutralizing antibodies in humans and have shown satisfactory outcomes in clinical applications [226]. In contrast, preexisting immunity to oncolytic viruses has been shown to potentiate immunotherapeutic efficacy [227].

Many clinical studies have revealed that CAR-T-cell therapy failure is associated with preexisting immunity to CAR-T cells carrying murine antigen receptors in some cases. Hence, patients may develop or already have anti-CAR antibodies that neutralize the binding of CAR-T cells to their targets, leading to their clearance and poor efficacy [228, 229]. Fully humanized single-chain variable fragment (scFv) of the antigen receptor, eliminating endogenous lymphocytes (lymphodepletion) by conditioning immunosuppressants and using newer generations of (CAR) T cells with non-scFv-based CARs are the strategies proposed to overcome the issue of anti-CAR antibodies [230–232]. In this context, standardized validated immunogenicity assays are proposed to be included in the flow chart of cellular therapies to ensure better assessment of immunogenicity concerns, thus improving overall outcomes [233, 234].

### Tumour resistance

#### The immunosuppressive tumour microenvironment

Extensive preclinical and clinical studies are underway to overcome and modulate the immunosuppressive TME that affects the efficacy of cancer vaccines and impairs T-cell functions. Many studies have revealed that using oncolytic virus vaccines aimed at transforming cold tumours into hot tumours results in better intratumoural T-cell infiltration and positive outcomes [235]. Moreover, vaccination with Newcastle disease virus (NDV) induces increased levels of systemic interferon-α with delayed tumour growth. It has also decreased the infiltration of myeloid-derived suppressor cells (MDSCs) [236, 237]. In the same regard, oncolytic viruses that deliver payloads targeting cancer agonists have been explored for their ability to enhance antitumour responses. One study revealed that using a modified oncolytic virus expressing a TGFβ inhibitor overcomes the tumour microenvironment that suppresses immune responses by increasing Treg cell fragility [238]. In addition, the use of oncolytic adenovirus expressing hyaluronidase results in a modified tumour matrix and potent antitumour effects with enhanced CD8 + cell infiltration in patients with metastatic pancreatic cancer [239, 240]. Similar results were shown using oncolytic VV encoding hyaluronidase in solid tumour murine models [241]. Investigations on the countering of stressful metabolic TMEs have also demonstrated that peroxisome proliferator activated receptor alpha (PPARα) agonists increase the efficacy of adenovirus chimpanzee (AdC68)-based melanoma cancer vaccines by allowing TILs to access glucose, thus increasing their ability to kill cancer cells [242]. These results were reproduced in a patient-derived xenograft (PDX) mouse model in which ex vivo-expanded TILs were treated with PPARα to improve their ability to slow the progression of autologous melanomas [243].

#### Resistance orchestrated by tumours and immune cells

One of the most upsetting and frustrating situations faced by the scientific community is the gradual loss of once-effective cancer therapy. Therefore, unravelling the mechanisms underlying resistance induced by inhibitor molecules expressed on tumours and immune cells is one of the major challenges that investigators must address to improve the clinical efficacy of cancer vaccines. Combining immune checkpoint inhibitors (ICIs), namely, anti-PD-1, anti-CTLA-4, anti-PD-L1, anti-LAG3 and anti-TIM3, with practically all developed cancer vaccines is a strategy employed in a large number of preclinical and clinical trials that have already yielded promising preliminary outcomes, driving researchers to continue this remarkable progress [244] (Table 5**).**

**Table 5:** Selected recent ongoing clinical trials of ICIs combined with cancer vaccines

Clinical Trial ID	Target	Indication	Phase	Current status and completion date
NCT05727904	Lifileucel (TILs therapy) plus Pembrolizumab (anti-PD-1)	Untreated advanced melanoma	III	Recruiting, 2030
NCT04217473	Oncolytic adenovirus TILT-123 in association with T-cell therapy with TILs	Advanced melanoma	I	Active, not recruiting, 2024
NCT05271318	Oncolytic adenovirus (TILT-123) plus pembrolizumab (anti-PD-1)	Ovarian Cancer	I	Recruiting, 2026
NCT05222932	oncolytic adenovirus TILT-123 in combination with avelumab (anti-PD-L1)	Solid Tumours	I	Recruiting, 2026
NCT03897881	Personalized cancer vaccine mRNA-4157 plus pembrolizumab (anti-PD-1)	High-risk melanoma	II	Recruiting, 2029
NCT05761717	mRNA personalized tumour vaccine combined with sintilimab (anti-PD-1)	Liver cancer	NA	Not yet recruiting, 2025
NCT05101356	Antineoplastic vaccine labvax 3(22)−23 plus pembrolizumab (anti-PD-1)	Advanced stage adenocarcinoma	I/II	Recruiting, 2030
NCT06324240	Tumour membrane vesicle vaccine plus ipilimumab (anti-CTLA-4)	Triple-negative breast cancer	I	Not yet recruiting, 2026
NCT06329908	Neo-DCVac plus PD1/PD-L1 inhibitors	Lung cancer	I	Recruiting, 2024
NCT03893903	IDH1R132H peptide vaccine plus avelumab (anti-PD-L1)	Progressive diffuse glioma	I	Active, not recruiting, 2024
NCT06218511	Peptide-based vaccine plus durvalumab (Anti-PD-L1)	Hepatocellular carcinoma	I	Recruiting, 2026
NCT05320081	CD30 CAR-T plus camrelizumab (anti-PD-1)	Lymphoma	II	Unknown status, 2024
NCT04995003	HER2 chimeric antigen receptor (CAR) T plus pembrolizumab	Advanced sarcoma	I	Recruiting, 2040
NCT05580354	BCG combined with tislelizumab (anti-PD-1)	Bladder cancer	IV	Not yet recruiting, 2025
NCT04397003	Neoantigen DNA vaccine Plus durvalumab (Anti-PD-L1)	Lung cancer	II	Recruiting, 2030

## Conclusions

Since the emergence of immune checkpoint inhibitor therapeutics in 2011, many other classes of these immunotherapies have been successively discovered, making dramatic and unprecedented contributions to the development of certain cancer therapies. Nevertheless, only a limited number of patients with certain malignancies benefit from their treatment. This could be explained by a lack of clinical efficacy and drug resistance in nonresponder patients. Because ICIs cannot be applied to the vast majority of cancer patients, alternative therapies have been sought as well, enabling the development of new cancer treatments, such as preventive and therapeutic cancer vaccines. Although the approval of the first cancer vaccine (BCG for bladder cancer) dates back to the 1990s, poor progress has been recorded in the field, and it was only after the development of genetic engineering, molecular biology and immunology techniques that we could truly witness major advancements in these therapies. Cancer vaccines have been a game changer in many cancer types and are considered the best alternatives to first-line treatments that fail to deliver satisfactory clinical outcomes.

However, cancer vaccines have their own limitations. Therefore, considerable work is being done to enhance their efficiency. Numerous strategies have been implemented to improve the overall outcomes of cancer vaccines and counteract resistance issues. Combinatorial treatments of cancer vaccines with ICIs, radiation and chemotherapy have provided potent antitumour immunity in patients, and accordingly, many of these combination therapies are being investigated in many ongoing clinical trials. Developing effective biomarkers and validated immune monitoring assays to assess vaccine efficacy and preexisting immunity could significantly enhance the success of these strategies. Moreover, machine learning tools, such as the 2024 Nobel Prize-winning artificial intelligence technology “AlphaFold,” are set to revolutionize vaccine design by accurately predicting the molecular structures of surface proteins. This advancement will facilitate improved identification of B-cell and T-cell epitopes, promising a brighter future for cancer therapy.

## Data Availability

No datasets were generated or analysed during the current study.

## Abbreviations

**FDA**: Food and Drug Administration
**EMA**: European Medicines Agency
**CDSCO**: Central Drugs Standard Control Organization
**NCI**: National Cancer Institute
**APCs**: Antigens presenting cells
**DCs**: Dendritic cells
**TLRs**: Toll-like receptors
**Th**: T helper
**CTLs**: Cytotoxic T lymphocytes
**PRFNs**: Perforins
**GRNZs**: Granzymes
**ADCC**: Antibody-dependent cellular toxicity
**TME**: Tumour microenvironment
**EMT**: Epithelial to mesenchymal transition
**NK**: Natural killers
**IBD**: Inflammatory bowel disease
**TDLN**: Tumour draining lymph node
**TAAs**: Tumour-associated antigens
**TSAs**: Tumour-specific antigens
**PeptiCRAd**: Peptide-coated conditionally replicating adenovirus
**CTLA4**: Cytotoxic T lymphocytes antigen 4
**PD-L1**: Programmed cell death ligand 1
**TIM3**: T-cell immunoglobulin mucin 3
**ICIs**: Immune checkpoint inhibitors
